# Macrophage Migration Inhibitory Factor -173 G/C Polymorphism: A Global Meta-Analysis across the Disease Spectrum

**DOI:** 10.3389/fgene.2018.00055

**Published:** 2018-03-01

**Authors:** Oscar Illescas, Juan C. Gomez-Verjan, Lizbeth García-Velázquez, Tzipe Govezensky, Miriam Rodriguez-Sosa

**Affiliations:** ^1^Unidad de Biomedicina, Facultad de Estudios Superiores-Iztacala, Universidad Nacional Autónoma de México, Tlalnepantla de Baz, Mexico; ^2^División de Investigación Básica, Instituto Nacional de Geriatría, Mexico City, Mexico; ^3^Departamento de Medicina Genómica y Toxicología Ambiental, Instituto de Investigaciones Biomédicas, Universidad Nacional Autónoma de México, Mexico City, Mexico; ^4^Departamento de Biología Molecular, Instituto de Investigaciones Biomédicas, Universidad Nacional Autónoma de México, Mexico City, Mexico

**Keywords:** meta-analysis, MIF, inflammation, autoimmune, age-related

## Abstract

Human macrophage migration inhibitory factor (MIF) is a cytokine that plays a role in several metabolic and inflammatory processes. Single nucleotide polymorphism (SNP) -173 G/C (rs755622) on *MIF* gene has been associated with numerous diseases, such as arthritis and cancer. However, most of the reports concerning the association of MIF with these and other pathologies are inconsistent and remain quite controversial. Therefore, we performed a meta-analysis from 96 case-control studies on -173 G/C *MIF* SNP and stratified the data according to the subjects geographic localization or the disease pathophysiology, in order to determine a more meaningful significance to this SNP. The polymorphism was strongly associated with an increased risk in autoimmune-inflammatory, infectious and age-related diseases on the dominant (OR: 0.74 [0.58–0.93], *P* < 0.01; OR: 0.81 [0.74–0.89], *P* < 0.0001; and OR: 0.81 [0.76–0.87], *P* < 0.0001, respectively) and the recessive models (OR: 0.74 [0.57–0.095], *P* < 0.01; OR: 0.66 [0.48–0.92], *P* < 0.0154; and OR: 0.70 [0.60–0.82], *P* < 0.0001, respectively). Also, significant association was found in the geographic localization setting for Asia, Europe and Latin America subdivisions in the dominant (OR: 0.76 [0.69–0.84], *P* < 0.0001; OR: 0.77 [0.72–0.83], *P* < 0.0001; OR: 0.61 [0.44–0.83], *P*-value: 0.0017, respectively) and overdominant models (OR: 0.85 [0.77–0.94], *P* < 0.0001; OR: 0.80 [0.75–0.86], *P* < 0.0001; OR: 0.73 [0.63–0.85], *P*-value: 0.0017, respectively). Afterwards, we implemented a network meta-analysis to compare the association of the polymorphism for two different subdivisions. We found a stronger association for autoimmune than for age-related or autoimmune-inflammatory diseases, and stronger association for infectious than for autoimmune-inflammatory diseases. We report for the first time a meta-analysis of rs755622 polymorphism with a variety of stratified diseases and populations. The study reveals a strong association of the polymorphism with autoimmune and infectious diseases. These results may help direct future research on *MIF*-173 G/C in diseases in which the relation is clearer and thus assist the search for more plausible applications.

## Introduction

Human macrophage migration inhibitory factor (MIF) is a cytokine involved in several metabolic and inflammatory processes, that have been widely studied in recent years. MIF is a versatile protein that acts as a potent upstream regulator of the immune system. This 12.5-kDa protein also plays an important role as a cytokine, chemokine, and even as an enzyme. As a cytokine, MIF has proinflammatory activities through the induction of the expression of other inflammatory cytokines and countering the effects of glucocorticoids (Calandra and Bucala, 1997). As a chemokine, it induces chemotaxis and arrests T and B lymphocytes, neutrophils and macrophages (Tillmann et al., 2013; Alampour-Rajabi et al., 2015). As an enzyme, MIF exhibits tautomerase and redox activities, which may be associated with the cytokine and chemokine functions of this protein (Nguyen et al., 2003a; Zhang et al., 2016).

The versatility of MIF is reflected on its roles in different endocrine and physiological systems, since it acts in several immunological, hormonal, metabolic and age-related pathways (Xia et al., 2015). For instance, the binding of MIF to the CD74 receptor, that is involved in different functions and the activation of several immune cells (Leng et al., 2003; Su et al., 2017), drives the activation of the MAP kinase ERK-1/2 signaling pathway (Mitchell et al., 1999), inducing production of inflammatory cytokines, such as TNFα, IL-1β, IL-6, IL-12, IL-8, IL-17, IFNγ, and prostaglandin E2 (Denkinger et al., 2004; Stojanović et al., 2009), and maintaining proinflammatory function and cell proliferation by inhibiting p53-dependent apoptosis (Hudson et al., 1999). Besides, this activity favors the expression of the cytokine receptors IL-1R and p55 TNFR (Toh et al., 2006). Moreover, MIF influences several processes that are important for the maintenance of cellular homeostasis and may influence the incidence and clinical manifestations of a variety of age-related manifestations associated with inflammation (Sauler et al., 2015). Notably, MIF has also been related with aging, since knocking out the *MIF* ortholog gene in mice has shown to extended their lifespan (Harper et al., 2010), and protect cells from oxidative stress-induced cell death (Nguyen et al., 2003b).

MIF characteristics of cell activation and proinflammatory action, make this molecule a constituent element of immunity and stress responses, contributing significantly to several immunopathologies resulting from excessive inflammation and autoimmunity (Donnelly and Bucala, 1997; Stosic-Grujicic et al., 2009), such as septic shock (Bernhagen et al., 1994), arthritis (Morand and Leech, 2005), diabetes (Sánchez-Zamora and Rodriguez-Sosa, 2014; Sánchez-Zamora et al., 2016), and other inflammatory autoimmune conditions (Denkinger et al., 2004; Morand, 2005; Santos and Morand, 2009). Also, the role of MIF in human disease has been recently emphasized since it has been suggested that polymorphisms modifying *MIF* expression could be associated with severe rheumatoid arthritis, fibrosis and asthma (Baugh et al., 2002; Donn et al., 2002; Mizue et al., 2005; Plant et al., 2005; Renner et al., 2005). In this context, polymorphisms of the *MIF* gene, located at chromosome 22q11.2, have generated increasing interest in clinical research, particularly the G/C SNP located at -173 (rs755622) in the *MIF* promoter sequence, which is the most widely studied variant in this gene. This polymorphism has been associated with the pathophysiology of arthritis, infections and inflammatory-related diseases (Donn et al., 2001; Baugh et al., 2002). The importance of this variant is due to its localization on the CpG island of the *MIF* promoter, where the G > C change generates an additional CpG motif and binding site for the transcription factor activator protein 4 (AP4) (Donn et al., 2001), which in turn increases the levels of both transcripts and protein (Matia-García et al., 2015; Ramayani et al., 2016; Bae and Lee, 2017).

Although, *MIF*-173 G/C SNP polymorphism has been associated with cancer (Tong et al., 2015b; Wang et al., 2015; Zhang et al., 2015), inflammatory bowel disease (IBD) (Hao et al., 2013; Shen et al., 2013; Zhang et al., 2013), arthritis (Lee et al., 2012; Xie et al., 2012; Bae and Lee, 2017), and renal disease (Tong et al., 2015a), in Asian and European populations the relation of the polymorphism with disease pathophysiology or geographical localization has not been clearly demonstrated, thus further investigation of the genetic associations of genes like MIF is important to improve the knowledge of the genetic basis of complex diseases, therefore we performed a comprehensive meta-analysis on the association of 96 case-control studies with geographical locations, pathophysiological signs and diseases. For the meta-analysis, cohorts were grouped in subdivisions according to four different settings. The geographic localization setting was based on the country origin of the individuals participating in each study, while the physiological localization setting considered the main organ or physiological system affected by the disease. The disease setting included arthritis, cancer and IBD, each representing a group of conditions with common characteristics, all previously evaluated in meta-analyses, but restricted to geographical areas such as Europe and Asia. The inclusion of the geographical localization setting was substantial for the study, considering that the polymorphism may not have the same association with disease in different populations, for example, the polymorphism has been clearly related to IBD in the Chinese population (Shen et al., 2013; Zhang et al., 2013), but it is not clear if this association is significant for other populations. Pathophysiology setting was divided according to clinical signs, such as the presence of a strong pathologic inflammatory component, a pathogenic autoimmune mechanism, or both. Age-related, composed by diseases frequently associated with aging, and infectious, composed by diseases of infectious origin, subdivisions were also included in this setting.

The aim of the present study was to generate a more meaningful conclusion on the association of *MIF*-173 G/C polymorphism with the genesis and progression of several diseases, and to examine whether this relation is stronger for populations from different geographical regions and/or diseases. To our knowledge, this is the first time that disease pathophysiological signs are used to perform a meta-analysis in order to explore its association with a polymorphism, and the first time that a simple network meta-analysis is used to compare and give importance to the associations of the polymorphism among diseases and disease subdivisions.

## Materials and methods

### Study search and selection

**Figure 1:**
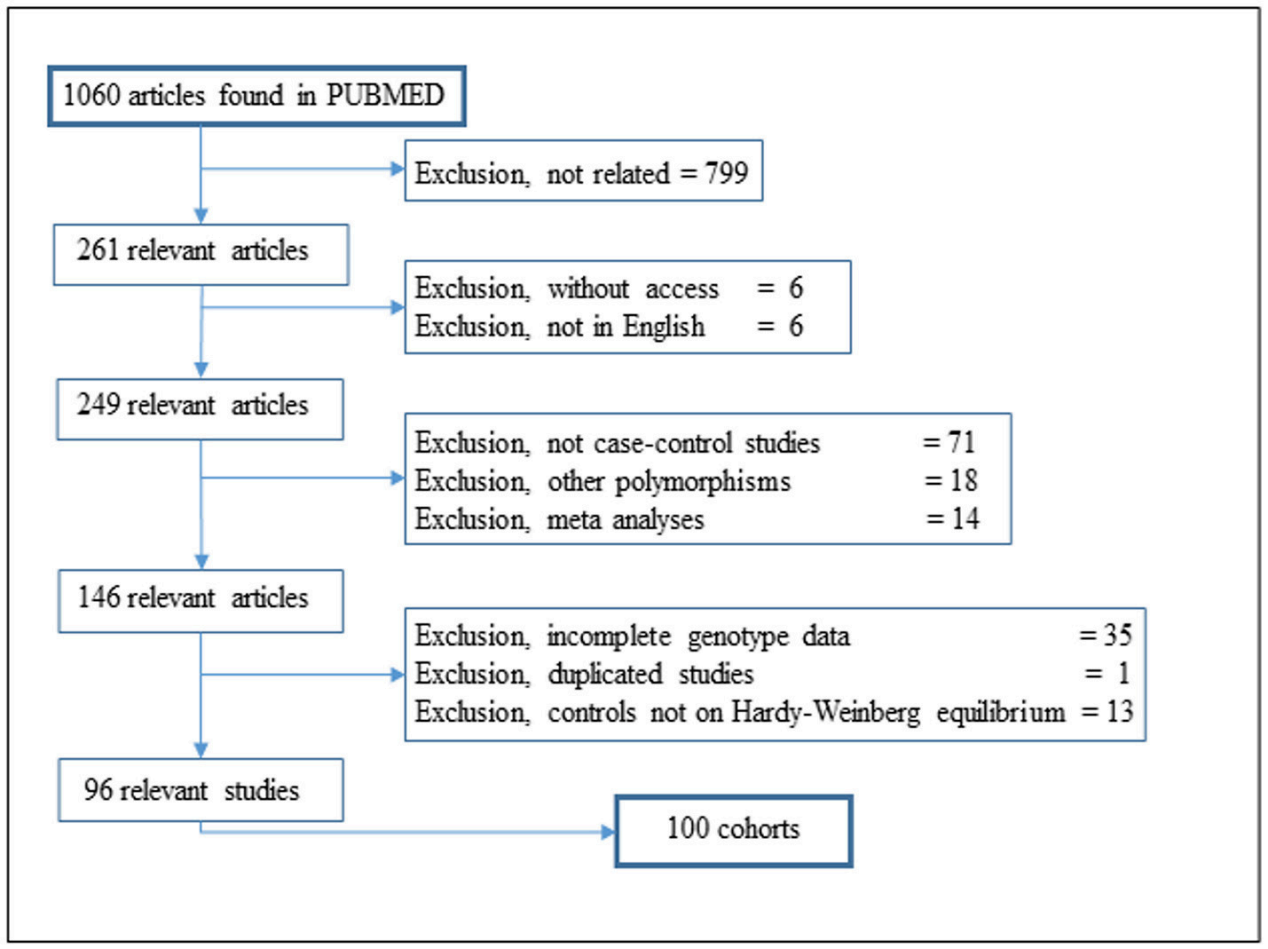
Flow diagram of article selection for the meta-analysis.

We searched for reports on the *MIF* polymorphism rs755622 in the Pubmed database using the search terms “MIF polymorphism,” “rs755622” and “*MIF*-173 G/C,” without any filter, and also the “MIF polymorphism” in Pubmed Commons. All searches were performed in March 2017. A total of 1,060 different studies were identified, and among these studies, 799 studies were not related after reading the abstracts, six studies lacked access to the full report and six studies were only available in a language different from English. No additional studies were identified in the references of the selected articles (Figure 1).

The inclusion criteria were: (1) Studies reporting the frequency of polymorphism rs755622, and (2) studies presenting both patients and control group data.

The exclusion criteria were: (1) Studies with genotype frequency data not included or incomplete, (2) reports that had self-contradictory data, and (3) studies with control data not in Hardy-Weinberg equilibrium.

Only articles meeting the inclusion criteria were included. Different studies from the same authors were compared to discard data duplications, and when a duplicate was observed or highly suspected, only the data from the earlier report was considered. Partially duplicated studies where not excluded from the meta-analysis when the duplication was restricted to the controls, as the comparison made in the meta-analysis is patients vs. controls and not controls vs. controls. However, these studies were not considered for the geographical distribution studies.

The data involved patients and healthy individual control groups, and the data from each single study were considered to be a cohort, a group of individuals sharing a defining characteristic. When two or more subtypes of a disease were reported, the data were merged into a single cohort. On the other hand, when two or more unrelated diseases were reported in the same study or the data were clearly divided between two or more countries, data were managed as two different cohorts.

Cohorts were grouped in subdivisions according to four different settings, all subdivisions include at least five different cohorts (Table 1). The geographic localization setting was based on the country origin of the individuals participating in each study. To evaluate if the relation of the polymorphism with disease was restricted to certain organs, the physiological localization setting considered the main organ or physiological system affected by the disease. The pathophysiology setting was divided according to common clinical signs, such as the presence of a strong pathologic inflammatory component, a pathogenic autoimmune mechanism, or both. Finally, the disease setting included arthritis, cancer and IBD, other diseases with < 5 cohorts were not included. If a cohort presented characteristics corresponding to more than one subdivision in the same setting, the cohort was included in both subdivisions. For instance, arthritis was included in both Age-related and Autoimmune-Inflammatory subdivisions.

**Table 1:** Composition of the subdivision settings.

**Setting**	**Subdivision**	**Components (disease or country)**
Disease	Arthritis	Arthritis, Ankylosing spondylitis, Rheumatic fever, Rheumatic heart disease, Psoriatic arthritis
	Cancer	Bladder cancer, Cervical cancer, Gastric cancer, Leukemia, Hamartoma
	IBD	Crohn's disease, Ulcerative colitis
Pathophysiology	Age-related	Alzheimer, Arthritis, Bladder cancer, Breast cancer, Cervical cancer, Coronary heart disease, Diabetes and obesity, Gastric cancer, Myocardial infarction, Obesity, Pancreatitis, Pneumococcal meningitis, Pneumonia, Post-surgery cardiac inflammation, Prostate cancer, Psoriatic arthritis, Sepsis
	Autoimmune	Asthma, Atopic dermatitis, Atopy, Autoimmune liver disease, Celiac disease, Autoimmune hepatitis, Lupus, Multiple sclerosis, Purpura, Scleroderma, VKH syndrome
	Autoimmune–Inflammatory	Arthritis, Ankylosing spondylitis, Behcet's disease, Lofgren syndrome, Psoriasis, Psoriatic arthritis, Rheumatic heart disease, Still's disease, Graves' disease
	Infectious	Chagas, Dengue, Hepatitis B, Leishmaniasis, Leprosy, Malaria, Pneumococcal meningitis, Pneumonia, Sepsis, Tuberculosis
	Inflammatory	Coronary heart disease, Gastritis, Cutaneous vasculitis, Crohn's disease, Ulcerative colitis, Post-surgery cardiac inflammation, Kawasaki disease, Pancreatitis, Peptic ulcer, Rheumatic fever
Geographic localization	Africa	Kenya, Egypt, Morocco, Zambia
	Asia	Japan, Korea, China, India, Indonesia
	Europe	Germany, UK, Italy, Netherlands, Poland, Switzerland, Spain, Russia, Czech Republic, Greece
	Latin America	Brazil, Colombia, Mexico, Peru
	Middle East	Iran, Saudi Arabia, Turkey
	North America	USA

*The diseases and countries considered for the stratification are listed beside each subdivision*.

### Statistical analyses

Deviation from HWE for each study was evaluated to improve the design and quality of the analysis. The proportions of genotypes CC, CG and GG were considered to be *p*^2^, 2*p*(1-*p*), and (1-*p*)^2^, respectively. The Chi-square test was estimated considering one degree of freedom and a *P* < 0.5.

We conducted the protocol for meta-analysis according to Lee *et al* (Lee, 2015). We also applied five different genetic models, Dominant (GG vs. CC + GC), Recessive (GC + GG vs. CC), Allelic (G vs. C), Homozygous (GG vs. CC) and Overdominant (GG + CC vs. GC). Data corresponding to patient and control sample size of each cohort is detailed in Table S1.

Hierarchical clustering heat-maps separated by k-means were performed with “heatmap” package (Perry, 2017) of the Rstudio software using R (Version 3.4) (RFfSC, 2017) and used to determine whether the cohorts could be grouped together in subdivisions according to the proposed geographical and physiological localization settings. Disease and pathophysiological settings were not presented as heat-maps, since we considered that their common clinical and pathological signs were sufficient information to validate the proposed subdivision.

In the present study, the OR and 95% CI were used to investigate the effect strength of the association between the *MIF*-173 G/C gene polymorphism and susceptibility to diverse diseases categorized according to multiple settings, including the geographical and physiological localizations, groups of related diseases, or their common pathophysiology. The results were considered significant when the *P*-values for the OR were below 0.05 (Tables 2, 3).

**Table 2:** Summary of the overall and all subdivisions meta-analysis results on the dominant, recessive, and allelic models.

**Setting**	**Subdivision**	**Cohorts**	**Genetic models**								
			**Dominant (GG vs. GC+CC)**			**Recessive (GG+GC vs. CC)**			**Alleles (G vs. C)**		
			**OR**	***P* val OR**	***P* val Het**	**OR**	***P* val OR**	***P* val Het**	**OR**	***P* val OR**	***P* val Het**
All studies		100	**0.80** **(0.76–0.85)**	**<0.0001**	**<0.0001**	**0.70** **(0.61–0.80)**	**<0.0001**	**0.013**	**0.78** **(0.73–0.84)**	**<0.0001**	**0.0626**
Disease	Arthritis	13	**0.70** **(0.49–0.99)**	**0.0437**	**<0.0001**	0.60 (0.35–1.03)	0.0631	0.0216	0.73 (0.55–0.96)	0.0229	0.0786
	Cancer	8	**0.85 (0.7–0.96)**	**0.0076**	**0.0005**	0.69 (0.54–0.89)	0.0054	0.462	0.84 (0.66–1.07)	0.1556	0.8338
	IBD	6	**0.88** **(0.73–1.07)**	0.206	0.1387	0.91 (0.69–1.21)	0.5243	0.258	0.88 (0.66–1.16)	0.3675	0.7025
Pathophysiology	Age-related	26	**0.81** **(0.76–0.87)**	**<0.0001**	**<0.0001**	**0.70** **(0.60–0.82)**	**<0.0001**	**0.0484**	0.77 (0.68–0.89)	0.0002	0.1891
	Autoimmune	18	0.85 (0.72–1.01)	0.0713	<0.0001	**0.63** **(0.51–0.78)**	**<0.0001**	**0.0411**	0.83 (0.70–0.99)	0.0376	0.572
	Autoimmune-inflammatory	17	**0.74** **(0.58–0.93)**	**0.0108**	**<0.0001**	**0.74** **(0.57–0.95)**	**0.0172**	**0.0209**	0.78 (0.65–0.93)	0.0056	0.2287
	Infectious	18	**0.81** **(0.74–0.89)**	**<0.0001**	**0.0073**	**0.66** **(0.48–0.92)**	**0.0154**	**0.0004**	0.76 (0.65–0.89)	0.0006	0.2022
	Inflammatory	16	0.78 (0.65–0.93)	0.006	0.0037	**0.69** **(0.56–0.85)**	**0.0006**	**0.0124**	0.74 (0.62–0.88)	0.0007	0.4566
Geographic localization	Africa	5	0.87 (0.53–1.44)	0.5938	0.0558	0.73 (0.36–1.48)	0.3775	0.0031	0.82 (0.49–1.38)	0.4557	0.0062
	Asia	35	**0.76** **(0.69–0.84)**	**<0.0001**	**<0.0001**	0.84 (0.64–1.12)	0.2372	0.9153	0.77 (0.69–0.87)	<0.0001	0.9767
	Europe	29	**0.77** **(0.72–0.83)**	**<0.0001**	**0.006**	**0.77** **(0.64–0.93)**	**0.0071**	**0.0914**	0.81 (0.71–0.94)	0.0044	0.9133
	Latin America	10	**0.61** **(0.44–0.83)**	**0.0017**	**0.004**	0.61 (0.46–0.83)	0.0012	0.3754	0.68 (0.56–0.83)	0.0002	0.516
	Middle East	14	0.93 (0.71–1.23)	0.6234	<0.0001	0.76 (0.38–1.53)	0.4462	<0.0001	**0.78** **(0.65–0.95)**	**0.0122**	**0.0138**
	North America	6	1.10 (0.99–1.23)	0.0789	0.034	0.84 (0.64–1.12)	0.2237	0.9153	1.13 (0.85–1.51)	0.4071	0.8811

*OR, Odds ratio; Pval, P-value; Het, Heterogeneity; IBD, Inflammatory bowel disease. All OR values have a 95% confidence interval. Results denoting a significant association are presented in bold*.

**Table 3:** Summary of the overall and all subdivisions meta-analysis results on homozygous and overdominant models.

**Setting**	**Subdivision**	**Cohorts**	**Genetic models**					
			**Homozygous (GG vs. CC)**			**Overdominant (GG+CC vs. GC)**		
			**OR**	***P* val OR**	***P* val Het**	**OR**	***P* val OR**	***P* val Het**
All studies		100	**0.63 (0.54–0.72)**	**<0.0001**	**0.0044**	**0.89 (0.84–0.95)**	**0.0003**	**0.0004**
Disease	Arthritis	13	**0.50** **(0.27–0.93)**	**0.028**	**0.0064**	0.76 (0.55–1.05)	0.0918	<0.0001
	Cancer	8	0.64 (0.49–0.85)	0.0016	0.1912	0.92 (0.82–1.04)	0.2052	0.0023
	IBD	6	0.67 (0.45–0.10)	0.053	0.6282	0.93 (0.78–1.11)	0.4592	0.1005
Pathophysiology	Age-related	26	**0.65 (0.55–0.77)**	**<0.0001**	**0.007**	0.86 (0.73–1.01)	0.0578	<0.0001
	Autoimmune	18	**0.58 (0.40–0.84)**	**0.0041**	**0.0078**	0.95 (0.81–1.11)	0.4893	<0.0001
	Autoimmune-inflammatory	17	**0.63 (0.42–0.96)**	**0.0306**	**0.0036**	**0.77 (0.63–0.93)**	**0.0074**	**<0.0001**
	Infectious	18	**0.63 (0.44–0.90)**	**0.0109**	**0.0007**	**0.88 (0.80–0.96)**	**0.006**	**0.038**
	Inflammatory	16	0.52 (0.40–0.67)	<0.0001	0.13333	0.90 (0.81–1.01)	0.0626	0.0252
Geographic	Africa	5	0.68 (0.28–1.63)	0.3847	0.0033	0.94 (0.75–1.18)	0.5681	0.2685
localization	Asia	35	**0.58 (0.50–0.67)**	**<0.0001**	**0.0138**	**0.85 (0.77–0.94)**	**0.0011**	**<0.0001**
	Europe	29	0.68 (0.56–0.84)	0.0002	0.1189	**0.80** **(0.75–0.86)**	**<0.0001**	**0.0432**
	Latin America	10	0.53 (0.39–0.71)	<0.0001	0.244	**0.73 (0.63–0.85)**	**<0.0001**	**0.0004**
	Middle East	14	0.75 (0.36–1.58)	0.0122	<0.0001	1.00 (0.86–1.17)	0.953	0.0255
	North America	6	0.92 (0.69–1.23)	0.4071	0.72	**1.14 (1.02–1.28)**	**0.0226**	**0.036**

*OR, Odds ratio; Pval, P-value; Het, Heterogeneity; IBD, Inflammatory bowel disease. All OR values have a 95% confidence interval. Results denoting a significant association are presented in bold*.

We calculated the heterogeneity using χ^2^-tests based in the *Q*-test and the I-squared (I^2^) statistics test. The pooled effect size (OR and SMD) was assessed based in the random-effect model if heterogeneity was considered statistically significant (*I*^2^-value more than 50% and *P* < 0.10). If not, then the fixed-effect model was used. To evaluate the specific effects of ethnicity and study quality, we also performed a subgroup analysis of different specific effects.

All data meta-analyses were performed using different functions included in the “metafor” R package (Viechtbauer, 2010; RFfSC, 2017), performed in Rstudio (Version 3.4). Metafor package consists of a collection of functions that allow the calculation of different effects size or outcome measures, to perfom a meta-analysis. We followed Viechtbauer (Viechtbauer, 2010) methodology for meta-analysis, using default functions. Complete description of algorithm is found in http://www.metafor-project.org/doku.php/metafor. Holm-Bonferroni correction was performed following the standard procedure (McDonald, 2014), using 0.05 as the critical value for the test, and the number of components (diseases or countries) stratified for each subdivision (Table 1), was used as the corresponding number of tests; the corresponding results are presented in Table S2.

A network meta-analysis (NMA) was performed according to Bucher et al. (1997), in which the OR of subdivision “A” was divided by the OR of subdivision “B,” or vice versa. Considering that all values obtained were below 1, the lower OR was always used as the numerator and the higher as the denominator. The numerical value obtained was 1 when the subdivisions presented equal association to the polymorphism and lower than 1 if the subdivision in the numerator had a stronger association than the denominator. We considered only values ≤0.90 for discussion.

## Results

### Included studies and stratification settings

A total of 96 publications were considered for this analysis, comprising 100 different cohorts for the rs755622 SNP. The general information and data mined from each study are provided in Table S1. Hardy-Weinberg Equilibrium (HWE) analysis was individually conducted for all cohorts, showing 15 cohort control groups out of HWE, which were not included in further analyses.

**Figure 2:**
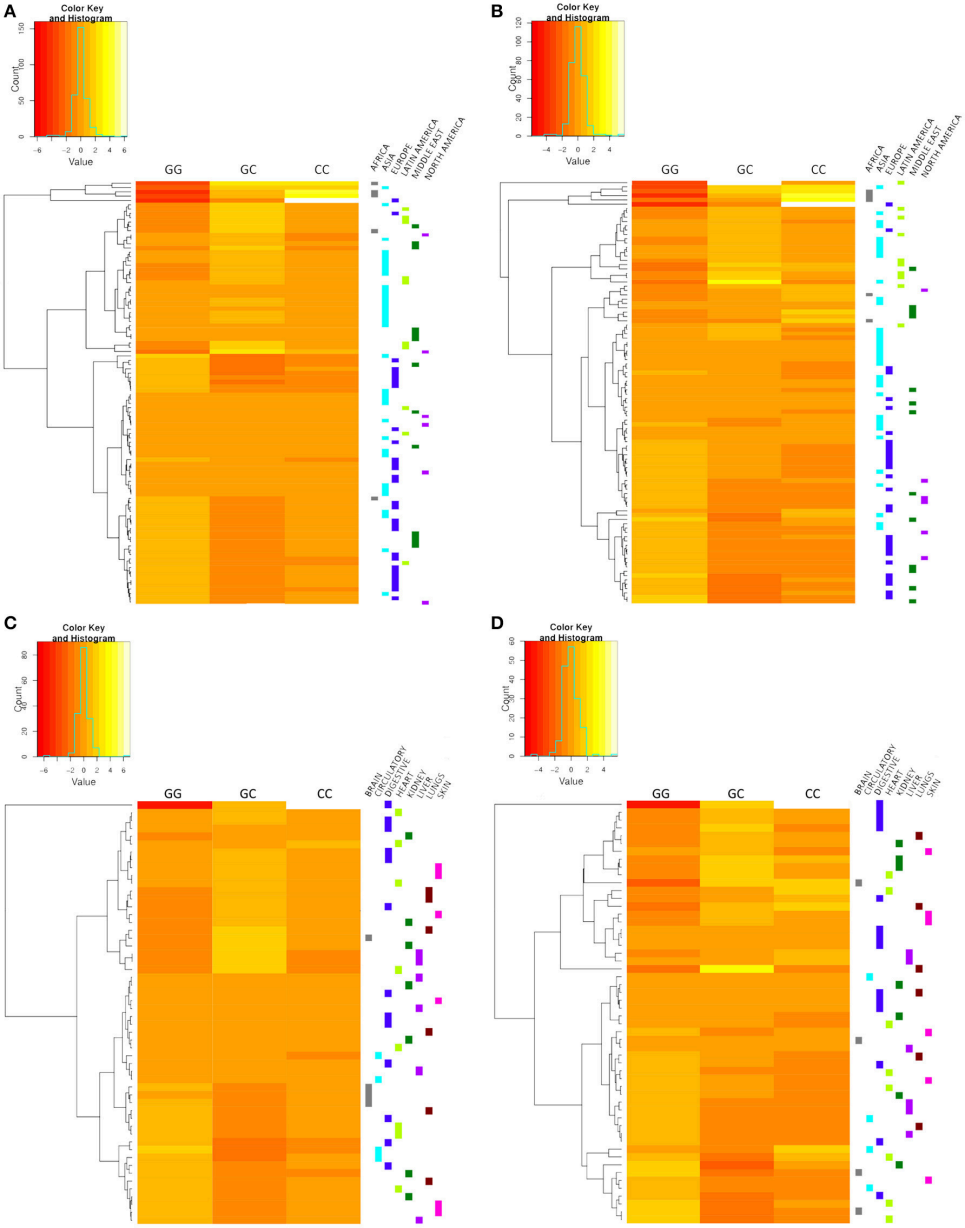
Hierarchical clustering heat-maps. Geographic localization setting controls **(A)** and patients **(B)**. Disease physiological localization setting controls **(C)** and patients **(D)**. The corresponding subdivision of each study is indicated on the right side of the heat-map, and each color represents a different subdivision. Cohorts of the European, Latin American, African, and North American subdivisions show a tendency to group together. No clear grouping was observed for the disease physiological localization setting.

The hierarchical clustering represented in heat-maps (Figure 2) indicated that cohorts from the same geographical subdivision, such as Europe and Latin America, generally tend to group together in both patients (Figure 2B) and controls (Figure 2A). High variability was observed in studies from Asia and the Middle East, in which there was no clear evidence of grouping this may be due to the variability of studies. Heatmaps corresponding to physiological localization setting (Figures 2C,D) showed no clustering, therefore, we did not continue the analysis of the physiological localization.

### Meta-analysis

The meta-analysis results are listed in Tables 2, 3. As results, subdivisions of the pathophysiology setting presented significant associations in 3 or 4 different models each. For example, the age-related subdivision was significantly associated in the dominant (OR: 0.81 CI:0.76–0.87, *P* < 0.0001, *P*-value Het: < 0.0001), recessive (OR: 0.70 CI:0.60–0.82, *P* < 0.0001, *P*-value Het: 0.0484), and homozygous (OR: 0.65 CI:0.55–0.77, *P* < 0.0001, *P*-value Het: 0.007) models.

Other diseases, such as infectious and autoimmune-inflammatory diseases, were significantly associated with the polymorphism in all of the genetic models, except for the allelic model. In the disease setting, only arthritis and cancer presented a significant association, the former in the dominant (OR: 0.70, CI:0.49–0.99, *P*-value: 0.0437, *P*-value Het: < 0.0001) and homozygous models (OR: 0.50 CI:0.27–0.93, *P*-value: 0.028, *P*-value Het: 0.0064) and the latter in the dominant model alone (OR: 0.85 CI:0.7–0.96, *P*-value: 0.0076, *P*-value Het: 0.0005). IBD subdivision was not significantly associated to the polymorphism in any subdivision.

In the geographic localization setting, Africa did not present a significant association in any subdivision, while Asia, Europe and Latin America presented a significant association in the dominant (Asia OR: 0.76 CI:0.69–0.84, *P* < 0.0001, *P*-value Het: < 0.0001), (Europe OR: 0.77 CI:0.72–0.83, *P* < 0.0001, *P*-value Het: 0.006), (Latin America OR: 0.61 CI:0.44–0.83, *P*-value: 0.0017, *P*-value Het: 0.004) and overdominant models (Asia OR: 0.85 CI:0.77–0.94, *P* < 0.0001, *P*-value Het: < 0.0001), (Europe OR: 0.80 CI:0.75–0.86, *P* < 0.0001, *P*-value Het: 0.006), (Latin America OR: 0.73 CI:0.63–0.85, *P*-value: 0.0017, *P*-value Het: 0.004).

**Figure 3:**
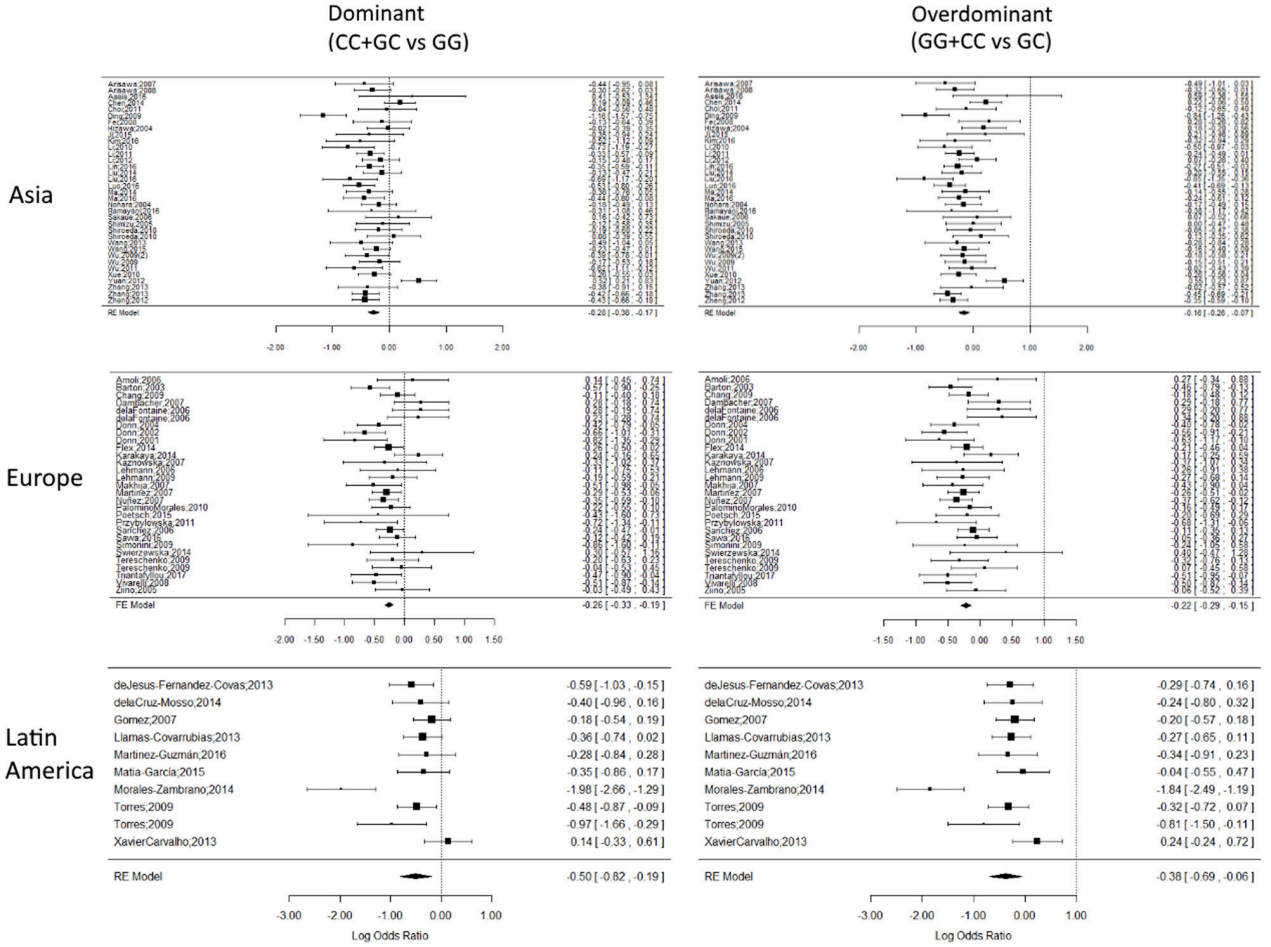
Representative forest plots. Forest plots of Asian, European and Latin American subdivisions on the dominant and overdominant models. The squares correspond to the study-specific OR, and the size reflects its weight. Horizontal lines correspond to the 95% CIs. The diamond represents the pooled ORs and 95% CIs of the overall population. The vertical solid line shows the Log OR of 0. There was no evidence of bias or the dominant effect of a single cohort over the global OR result.

Forest plots for representative subdivisions and genetic models are presented in Figure 3. Each subdivision and genetic model forest plots are presented in Figures S1–S3. Holm-Bonferroni correction test was performed to validate statistical significance on every model, the corrected significance is reported in Table S2, and coincides with most of the results presented in Tables 2, 3. Although subdivisions like arthritis, infectious and autoimmune-inflammatory ceased to be significantly associated in several of the gene models after the correction was applied.

### Funnel plots

**Figure 4:**
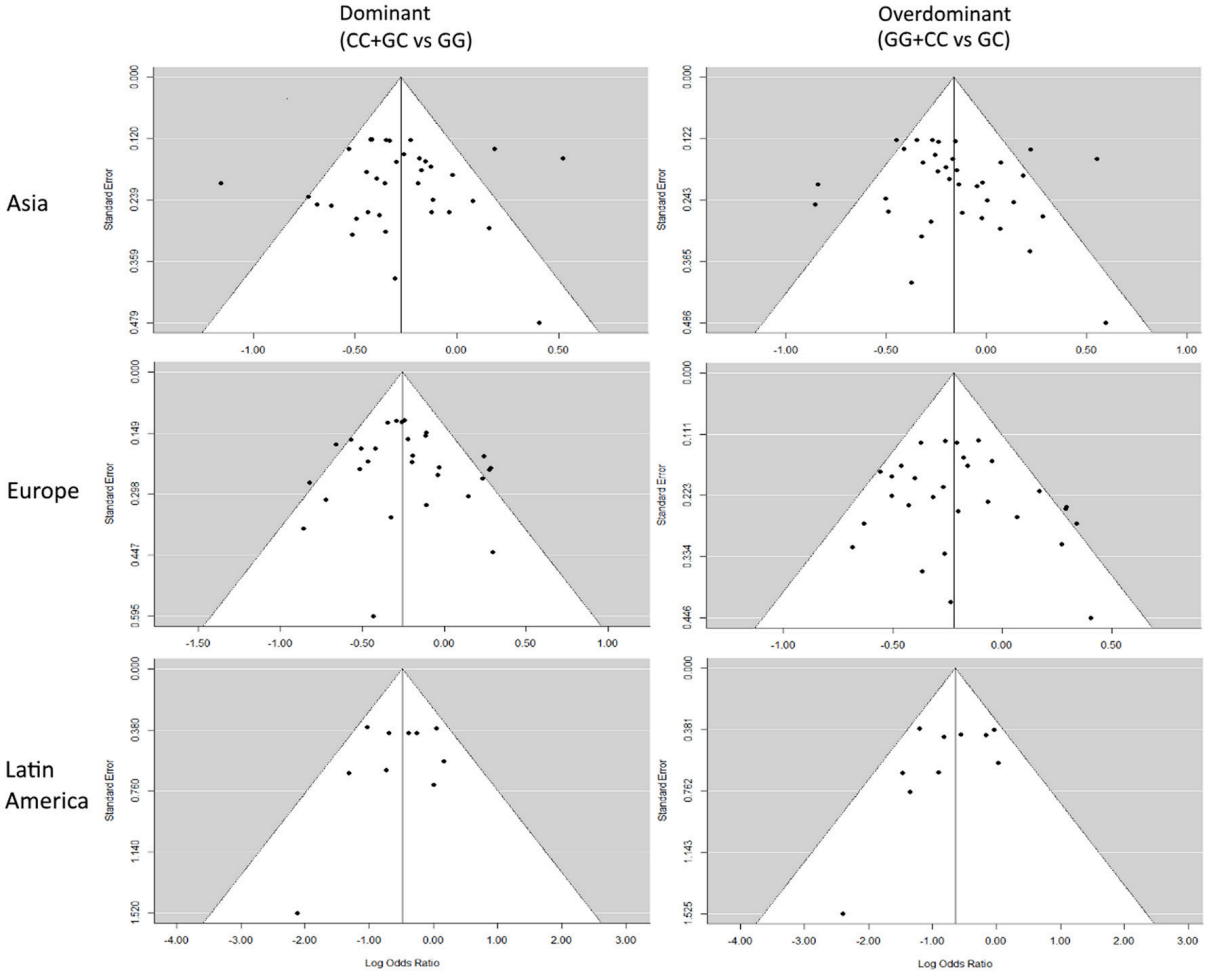
Representative Funnel plots. Funnel plots of Asian, European, and Latin American subdivisions on the dominant and overdominant models. The vertical solid line represents the summary effect estimate, and the 95% CI zone is shown in white.

Representative Funnel plots are presented in Figure 4. There was no evidence of bias in the Funnel plots, as most of the studies were observed to be inside the 95% confidence limit and the general distribution was symmetrical. The Funnel plots for every subdivision and genetic model analyzed are presented in Figures S4–S6.

The Funnel and forest plots corresponding to the entire meta-analysis in the five different genetic models are presented in Figure S7.

### Network meta-analysis

A NMA was performed using a simple method proposed by Bucher et al. (1997) that involves the relation of the OR of Effect A over the OR of Effect B. Here, we compared the OR of different subdivisions of the pathophysiological setting with the dominant, recessive and homozygous genetic models, which showed more significant association results in the meta-analysis. The results are presented in Table 4.

**Table 4:** Network meta-analysis according to the method proposed by Bucher et al. (1997).

**Network meta-analysis (Buchers)**									
**Subdivisions (a vs. b)**	**Dominant**			**Recessive**			**Homozygous**		
	**OR a**	**OR b**	**OR Bucher**	**OR a**	**OR b**	**OR Bucher**	**OR a**	**OR b**	**OR Bucher**
Age-related vs. Autoimmune	0.81	–	–	0.70	0.63	**0.90 (b/a)**	0.65	0.58	**0.89 (b/a)**
Age-related vs. A. I.	0.81	0.74	0.91 (b/a)	0.70	0.74	0.95 (a/b)	0.65	0.63	0.97 (b/a)
Age-related vs. Infectious	0.81	0.81	1.0	0.70	0.66	0.94 (b/a)	0.65	0.63	0.97 (b/a)
Age-related vs. Inflammatory	0.81	0.78	0.96 (b/a)	0.70	0.69	0.99 (b/a)	0.65	–	–
Autoimmune vs. A. I.	–	0.74	–	0.63	0.74	**0.85 (a/b)**	0.58	0.63	0.92 (a/b)
Autoimmune vs. Infectious	–	0.81	–	0.63	0.66	0.95 (a/b)	0.58	0.63	0.92 (a/b)
Autoimmune vs. Inflammatory	–	0.78	–	0.63	0.69	0.91 (a/b)	0.58	–	–
A. I. vs. Infectious	0.74	0.81	0.91 (a/b)	0.74	0.66	**0.89 (b/a)**	0.63	0.63	1.0
A. I. vs. Inflammatory	0.74	0.78	0.95 (a/b)	0.74	0.69	0.93 (b/a)	0.63	–	–
Infectious vs. Inflammatory	0.81	0.78	0.96 (b/a)	0.66	0.69	0.96 (a/b)	0.63	–	–

*Results below 0.9 are presented in bold. A.I, Autoimmune-inflammatory*.

### Geographic subdivisions genotypic frequency

**Figure 5:**
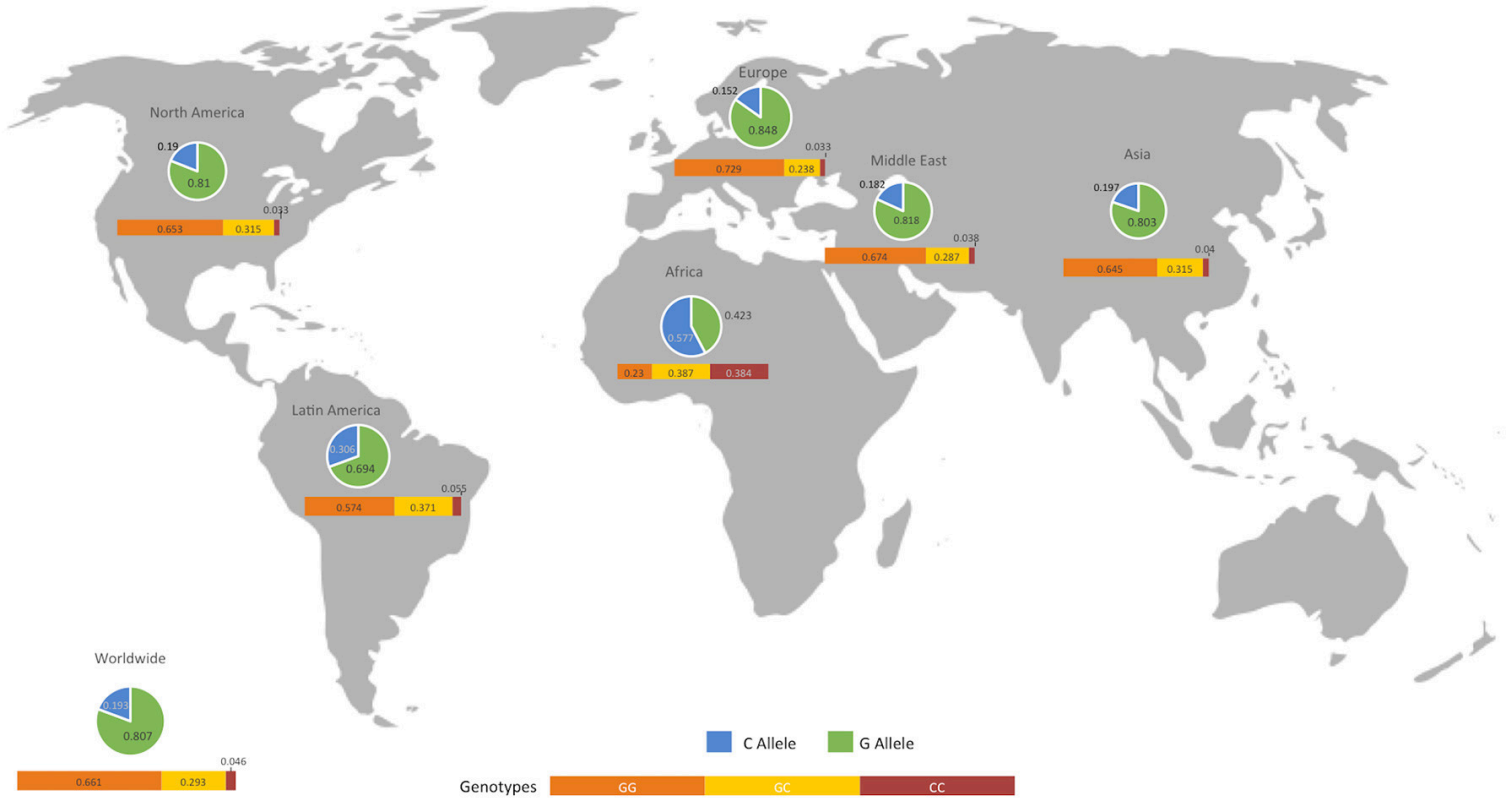
Geographical distribution of alleles and genotypes at the *MIF* -173 G/C SNP. The map shows the allelic and genotypic frequencies in control individuals from all the studies included in the analysis. An extensive comparison with patients and biological databases is described in Table 5.

We presented the frequencies of all of the studies included in the meta-analysis for both controls and patients, divided according to geographical setting (Figure 5, Table 5). We also compared the data obtained from the 1,000 genomes project (Consortium, 2015) with our results, as shown in Table 5.

**Table 5:** Comparison of the frequencies obtained in the present meta-analysis and those reported by the 1,000 genome project (Consortium, 2015).

**Zone**	**1000 genome project**					**Controls**			**Meta-analysis**				**Patients**		
	**GG**	**GC**	**CC**	**G**	**C**	**GG**	**GC**	**CC**	**G**	**C**	**GG**	**GC**	**CC**	**G**	**C**
Africa	0.31	0.511	0.179	0.566	0.434	0.23	0.387	0.384	0.423	0.577	0.224	0.407	0.369	0.428	0.572
Latin America	0.591	0.346	0.063	0.764	0.236	0.574	0.371	0.055	0.694	0.306	0.464	0.459	0.076	0.694	0.306
Asian	0.641	0.325	0.034	0.804	0.196	0.645	0.315	0.04	0.803	0.197	0.578	0.357	0.065	0.756	0.244
Europe	0.66	0.308	0.032	0.814	0.186	0.729	0.238	0.033	0.848	0.152	0.66	0.282	0.058	0.801	0.199
Middle East	–	–	–	–	–	0.674	0.287	0.038	0.818	0.182	0.655	0.285	0.061	0.797	0.203
North America	–	–	–	–	–	0.653	0.315	0.033	0.81	0.19	0.681	0.284	0.035	0.823	0.177
All	0.546	0.375	0.079	0.733	0.267	0.661	0.293	0.046	0.807	0.193	0.606	0.325	0.069	0.769	0.231

*There is no group in the 1000 genome project corresponding to the countries included in the Middle East and North American subdivisions*.

## Discussion

Genetic association studies are important for epidemiological analyses, as these studies can identify candidate genome regions that contribute or associate to specific diseases (Lee, 2015). In the present meta-analysis, 96 control-case studies comprising 100 different cohorts evaluating the relationship of the *MIF*-173 G/C polymorphism with a variety of diseases were included. Considering their significant diversity, these studies were categorized according to the determined common characteristics, such as the geographic localization or pathophysiology of the disease, instead of the classical comparison of overall results. Clustering analysis for the geographic setting showed a tendency to cluster studies of African, European, Latin American, and North American subdivisions. The Asian and Middle East subdivisions showed no cluster tendency, probably due to the admixture of the studies, nevertheless, we decided to maintain the complete setting in further analyses, in order to compare among different regions.

After the analysis of the different genetic models used in the meta-analysis, the allelic model showed less significant associations with the subdivisions, reflecting not only a lack of difference between controls and patients at allelic level but also a higher heterogeneity at the genotype level. We included this model in the analysis to determine if a strong influence of a single allele on disease was present, however, this was not the case. The localization of the polymorphism on the promoter sequence indicates that its biological significance is related to differences in the transcript expression levels (Matia-García et al., 2015; Ramayani et al., 2016; Bae and Lee, 2017), and this differences are better explained in an individual by genotypes than by alleles. The use of the allelic model duplicates the data available for analysis in comparison to genotypes, but does not provide useful information on the relation with disease, considering the nature of the polymorphism, and indicating that this model is not useful in our current analysis. The dominant model presented a higher number of significant correlations with subdivisions, with 9 out of 15 subdivisions, this model is defined by the presence (or absence) of the C allele in the genotype, indicating the importance of this allele association with diseases; however, this association only exists when genotypes, but not individual alleles, are considered. Holm-Bonferroni correction, performed to validate statistical significance on every model, also confirmed the significant association found in the models described.

Arthritis and cancer, two conditions previously associated with MIF and the -173 GC SNP, presented significant associations in at least one model, while IBD did not demonstrate any association. In this context, the influence of *MIF* polymorphisms on IBDs has previously been studied in other meta-analyses, including four published studies focused on Asian and European cohorts, and the results showed a clear association between the *MIF* -173 GC SNP and IBD (Hao et al., 2013; Shen et al., 2013; Zhang et al., 2013; Yang et al., 2016). However, we did not observe a significant correlation of the polymorphism with IBD likely due to the inclusion of studies from other geographic localization, this correlates with other meta-analyses that have explored whether the *MIF* -173 GC SNP is associated with an increased cancer risk, obtaining heterogeneous results. For instance, one study in Asian population reported a significant relation between this polymorphism and the risk of developing gastrointestinal cancer or hematological malignancy (Tong et al., 2015b), while another study observed a significant relation with prostate and non-solid cancers (Zhang et al., 2015), and a third study, limited to the Chinese population, observed a general significant relation, particularly with non-solid tumors (Wang et al., 2015). In this context, we analyzed all cancer studies together and observed a significant relation speacially in the dominant model, indicating an increased risk for GC or CC genotype carriers patients.

The relation of the polymorphism with arthritis has also been reported in meta-analyses, and a significant relation was observed, but the included studies were almost exclusively limited to European populations (Lee et al., 2012; Xie et al., 2012). In the present meta-analysis, we considered studies from all geographic localizations and determined that there was an association speacially in dominant and homozygous models. The results in the dominant model indicated an increased risk for GC or CC genotype carriers, while the results in the homozygous model confirmed a risk for CC genotype carriers.

In the present study, we observed an association between GC and CC genotypes and increased disease risk in the Asian, European and Latin American subdivisions, according to the results in the dominant and overdominant models, which were also supported by the results of the European subdivision in the recessive model and Asian subdivision in the homozygous model. These results indicate the importance of geographic localization and ethnic origin in disease even in the same country. For example, in the United States, the highest incidence and mortality of colorectal cancer is observed in non-Hispanic black people, while the lowest incidence is observed in the Asian-American/Pacific islander group (Siegel et al., 2017). Considering this, is important to indicate that there are other factors that may also contribute to these disparities between ethnicities, such as socioeconomic status, environmental conditions, genetic admixture or ancestry and epigenetic background in early development (Tobi et al., 2014). These results do not discriminate between different diseases and therefore should be cautiously considered.

We also compared the genotypic and allelic frequencies obtained in each geographic localization subdivision in the present meta-analysis with previously reported results. The allele frequencies of the African, Latin American, Asian and European subdivisions were consistent with those reported in the 1,000 genome project (Consortium, 2015). Notably, differences between patients and controls were apparently accentuated in genotypic frequencies and not as clear in allelic frequencies. This result is consistent with the low number of subdivisions associated with this polymorphism in the allelic model.

Another setting used to categorize the data was based in disease pathophysiology, that includes a subdivision composed of conditions with degenerative components or associated to age-related diseases. Considering the preponderant role of MIF in the immune response and in aging, we also included subdivisions composed of diseases characterized by a strong and pathologic inflammatory response, a pathogenic autoimmune process, or both. The results of the present meta-analysis showed a clear relation of the polymorphism with disease in this subdivision in the dominant, recessive and homozygous models, correlated with several studies exploring the role of MIF in homeostasis and inflammation, and since the pathogenesis of most age-related diseases led to a special type of chronic inflammation, the so called “inflammaging,” is therefore not surprising that we observed an association of the *MIF* polymorphism with these diseases (Harper et al., 2010; Palumbo et al., 2013; Sauler et al., 2015; Xu et al., 2016).

Infectious subdivision presented an association with the polymorphism in all genetic models but the allelic. The association was unexpected, considering that MIF has an effect in the expression of TLR4, the signal transducing molecule of the LPS receptor (Roger et al., 2003; Das et al., 2013). However, the subdivision included infectious diseases caused by etiologic agents of different kinds, not only Gram-negative bacteria. Interestingly, the association to the polymorphism with infectious diseases suggests that MIF expression may not be part of an effective immune response against infection. Zhong et al. (2005) proposed that the variations in the population distribution of alleles from *MIF* microsatellite polymorphism rs5844572, suggest the existence of a selective pressures acting on the *MIF* locus. In the case of rs5844572, that is also localized in *MIF* promoter sequence, the pressure of continuous exposition to infectious diseases would explain the high prevalence of low-expression *MIF* alleles in Africa. This proposition may not be applicable to the -173 GC polymorphism, considering that the frequency of *MIF* low-expression allele G is the same in patients and in controls, even though both are lower than the reported by the 1,000 genome project (Table 5).

Autoimmune-inflammatory and inflammatory subdivisions also presented significant associations indicating an increased risk for the GC and CC genotypes according to multiple models. Similarly, the autoimmune subdivision presented an increased risk for CC genotype carriers according to the recessive and homozygous models.

All of the subdivisions in the pathophysiology setting presented an increased risk for C allele carrier genotypes. Bucher indirect comparison is an NMA statistical method used to determine which of two different treatments is more efficient, comparing the magnitude of the treatment effect. Thus, we performed a simple NMA (Bucher et al., 1997) to compare the effect of the *MIF* -173 GC SNP on the disease risk for different subdivisions. In this case, we compared the association of the polymorphism with diseases in each subdivision and model of the pathophysiology setting, and the results indicate whether one subdivision has a greater effect on disease risk. To our knowledge, this study is the first to perform a NMA in a genetic association model. Our results indicate that in the recessive model, the autoimmune subdivision has a stronger association with the polymorphism than the age-related and autoimmune-inflammatory subdivisions. On the other hand, results indicate a stronger association of the infectious subdivision than the autoimmune-inflammatory subdivision. Thus, the polymorphism and its effect in the expression of the protein, have a significant role in the genesis or development of the diseases in the autoimmune and infectious subdivisions. Also, it indicates that even when age-related and autoimmune-inflammatory diseases may be associated to the polymorphism of MIF, this association is weaker, and less likely to be of biological interest.

After the Holm-Bonferroni correction was applied some subdivisions ceased to be significantly associated with disease. Arthritis subdivision lost significance in all models, while autoimmune-inflammatory subdivision was still significantly related to disease only in the overdominant model, and the infectious subdivision maintained it in the dominant and allelic models. It should be noted that subdivisions of the geographical localization setting majorly maintained its significant association with disease in different models, indicating a strong correlation with disease. These results should be considered carefully, as the Bonferroni test is highly conservative and stringent. Even though arthritis subdivision presented no significant association according to this correction, the relation between the diseases included in the subdivision remains true, as all of them are rheumatoid disorders, and may not be treated as different traits. Also, the test has limitations regarding the geographical localization setting, considering that different countries does not necessarily have different populations, and one country may have many.

In summary, our results indicated that the GC and CC genotypes are associated with arthritis, cancer, and importantly, all of the subdivisions proposed in the pathophysiology setting. The association of these genotypes with risk was also confirmed when the studies were stratified according to geographic localization. The global meta-analysis showed an association in all models, indicating the association of genotypes including the allele C, with the risk of developing different diseases. When comparing between subdivisions, the CC genotype had a stronger effect on the risk to develop diseases from the autoimmune and infectious subdivisions than those in the age-related and autoimmune-inflammatory subdivisions. Moreover, the allelic model did not provide significant information on this polymorphism, indicating that the influence on determined zones or diseases is not dependent on the presence of the C allele, but rather on the specific genotype. Limitations in the present study should be noted, as in any meta-analysis, being the principal limitation the low number of studies in certain subdivisions, such as African and North American. Another limitation to consider is that the Holm-Bonferroni correction applied to validate the statistical significance of the *P*-values is conservative and stringent, however, a stringent validation was needed, considering that the study proposes the inclusion of different traits in the same analysis. Nevertheless, the present study a meta-analysis including case-control studies of the relation of a wide variety of diseases with a polymorphism. Further experiments are required to confirm the relations found and detail the subyacent mechanisms, and this may help direct future research on *MIF*-173 G/C in diseases in which the relation is clearer and thus assist the search for more plausible applications.

## Author contributions

All of the authors significantly contributed to this work. OP and MR-S designed the study; OP conducted the study selection and data extraction; OP, JG-V, LG-V, and TG performed the data analysis, genetic models assessment and prepared the figures and tables; OP and MR-S drafted the manuscript; all authors revised it critically for important intellectual content and approved the final draft.

### Conflict of interest statement

The authors declare that the research was conducted in the absence of any commercial or financial relationships that could be construed as a potential conflict of interest.
