# Supplementary figures and images for: Macrophage Migration Inhibitory Factor -173 G/C Polymorphism: A Global Meta-Analysis across the Disease Spectrum
Source: Front Genet. 2018 Mar 1;9:55. doi: 10.3389/fgene.2018.00055 (PMC5839154; doi:10.3389/fgene.2018.00055)

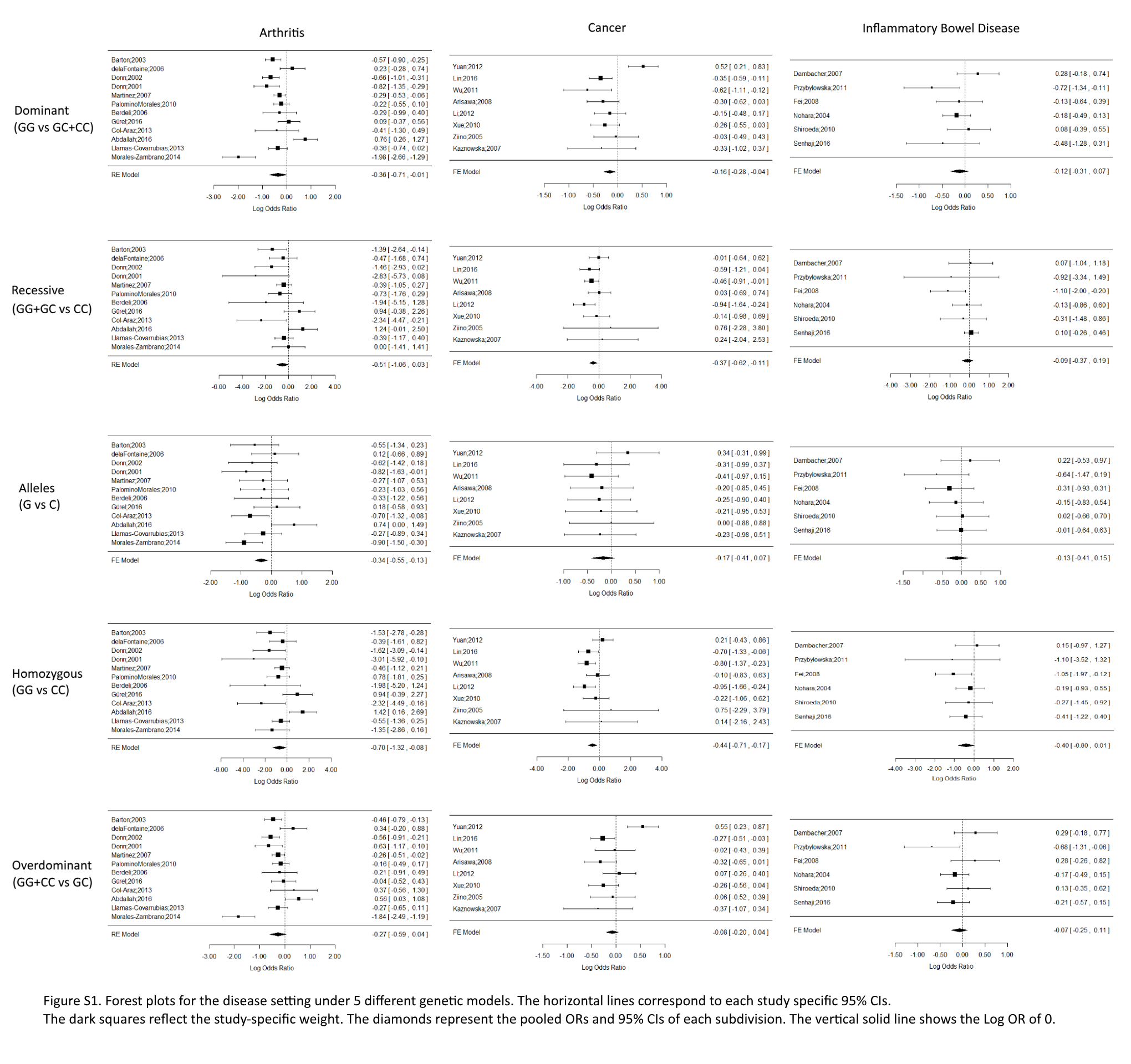

Supplement: Supplementary file 3 [file Image1.tif]

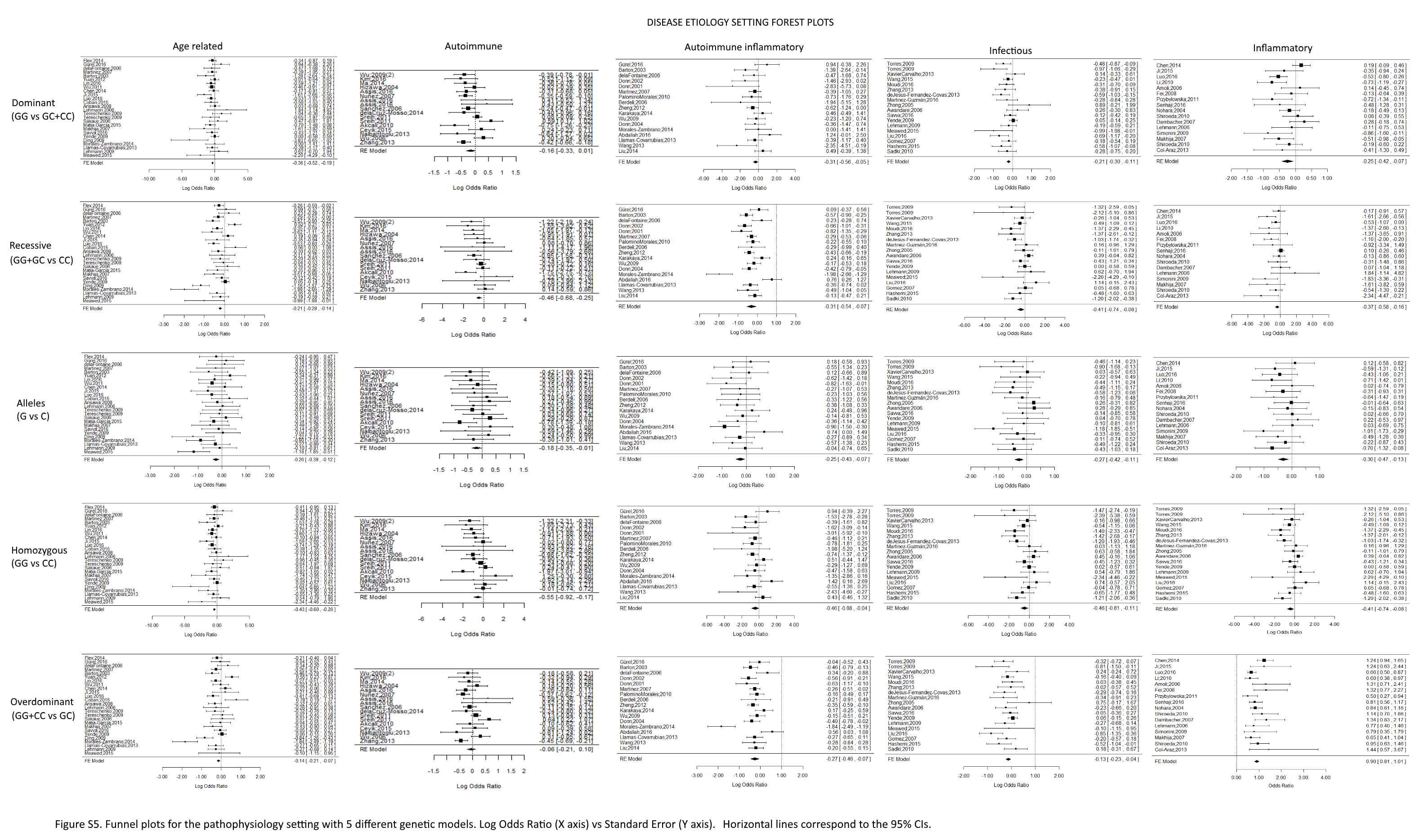

Supplement: Supplementary file 4 [file Image2.tif]

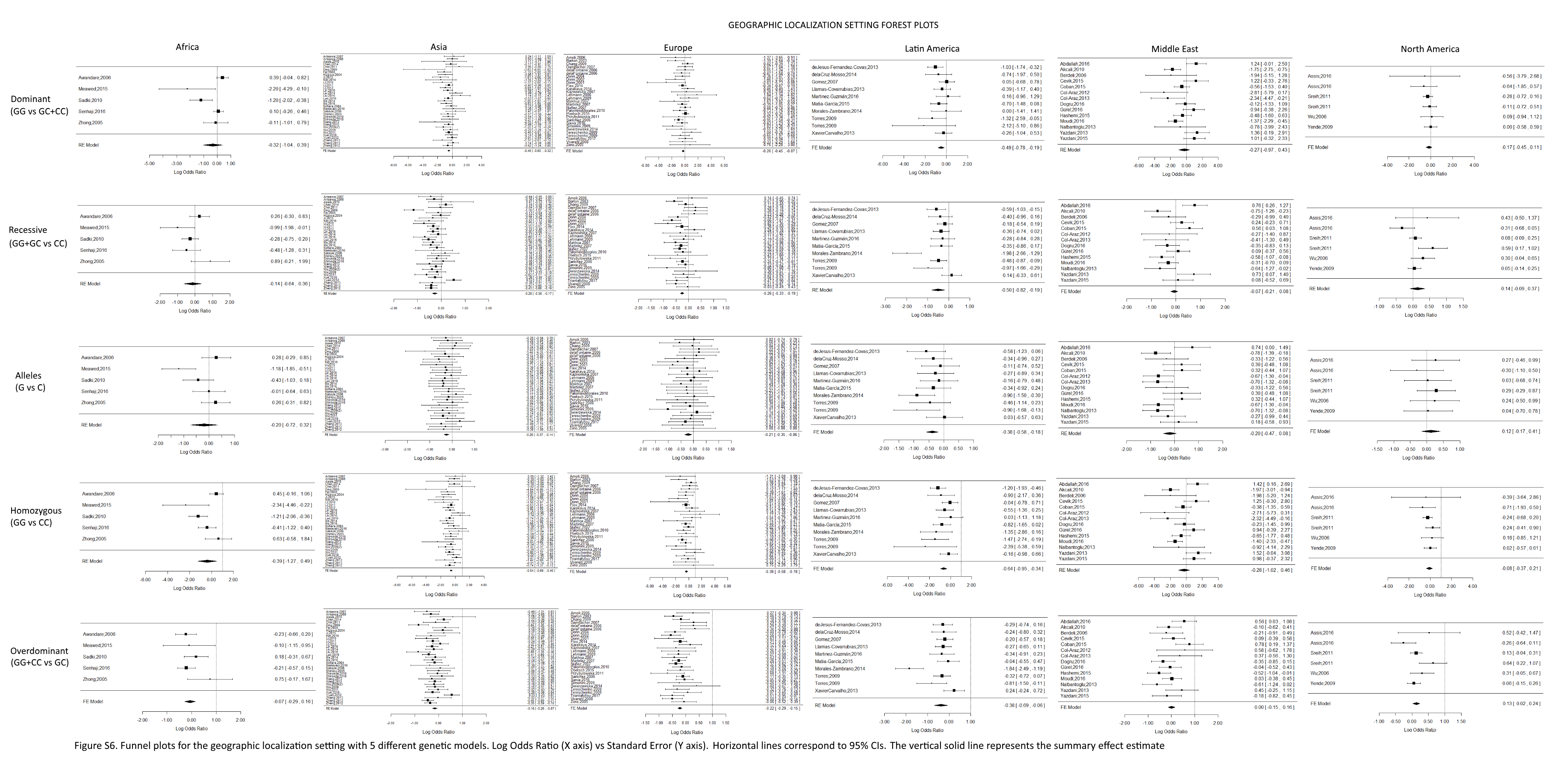

Supplement: Supplementary file 5 [file Image3.tif]

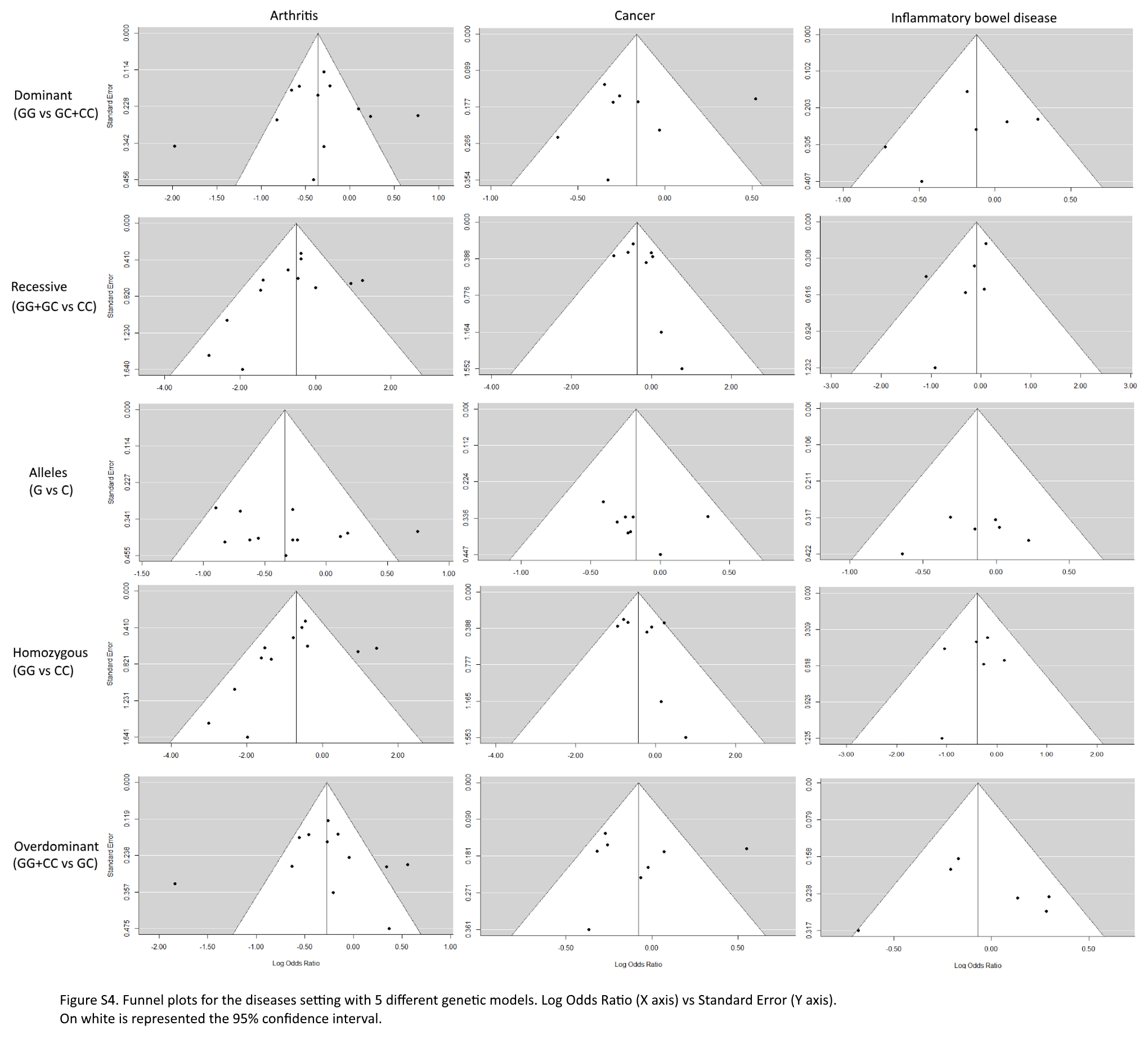

Supplement: Supplementary file 6 [file Image4.tif]

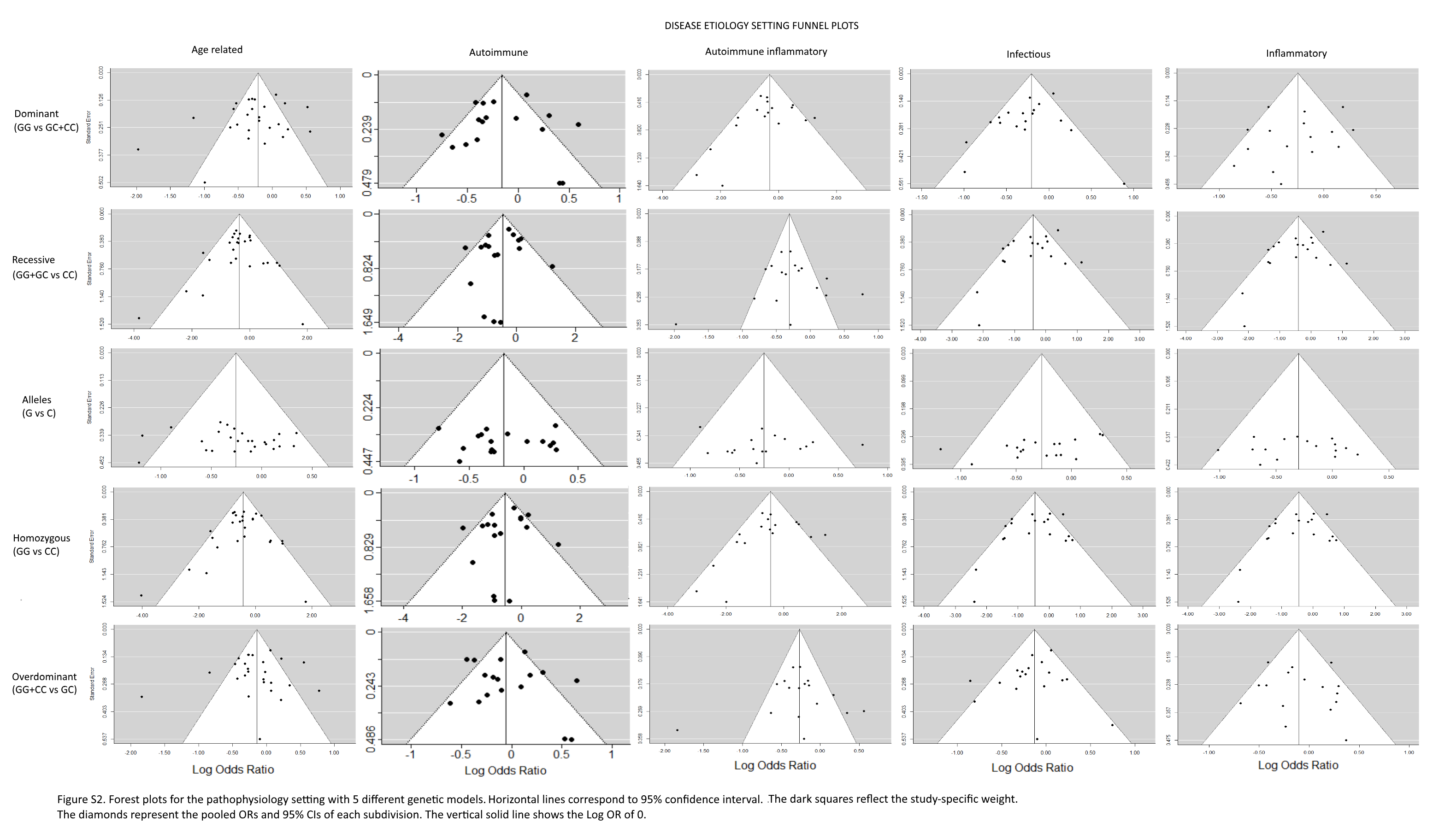

Supplement: Supplementary file 7 [file Image5.tif]

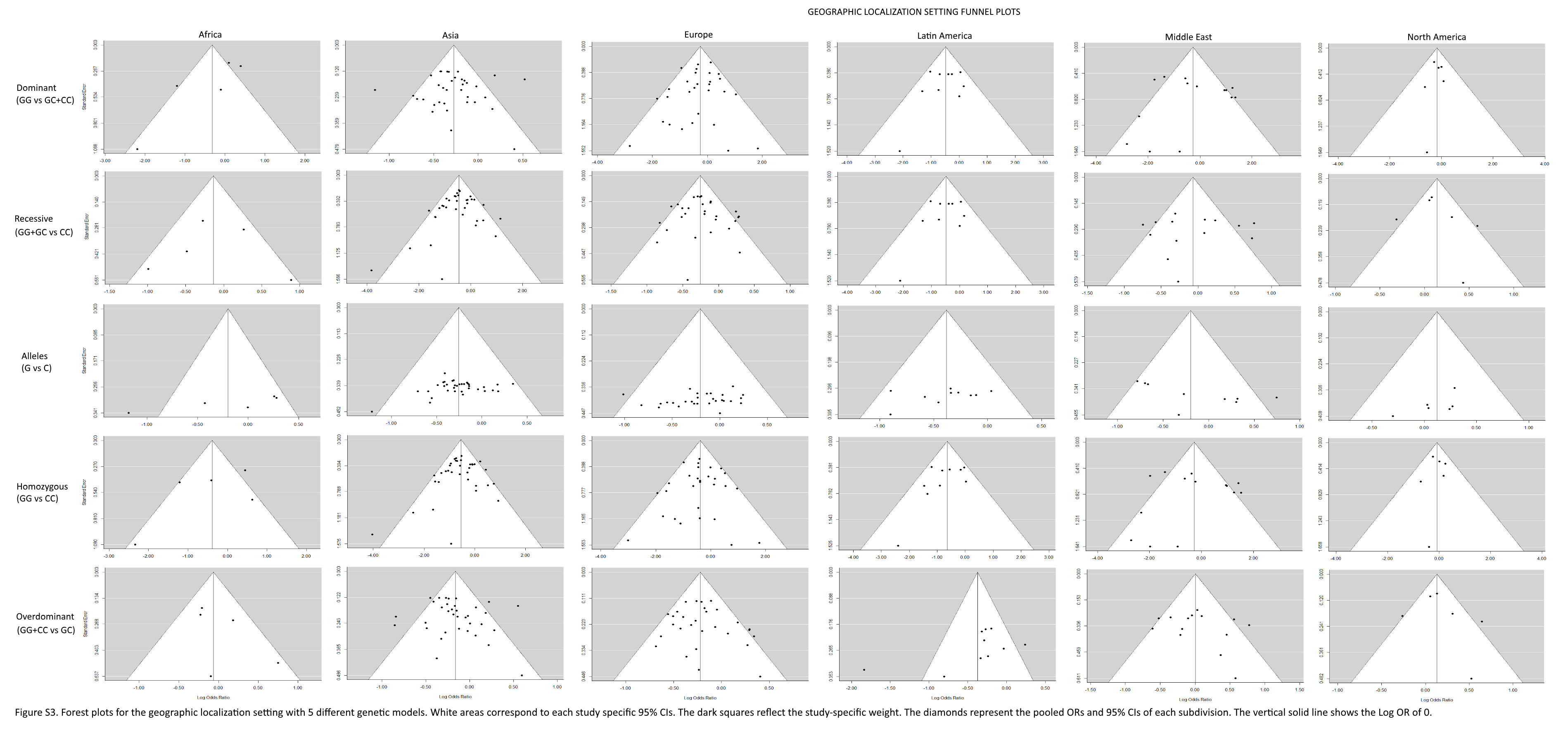

Supplement: Supplementary file 8 [file Image6.tif]

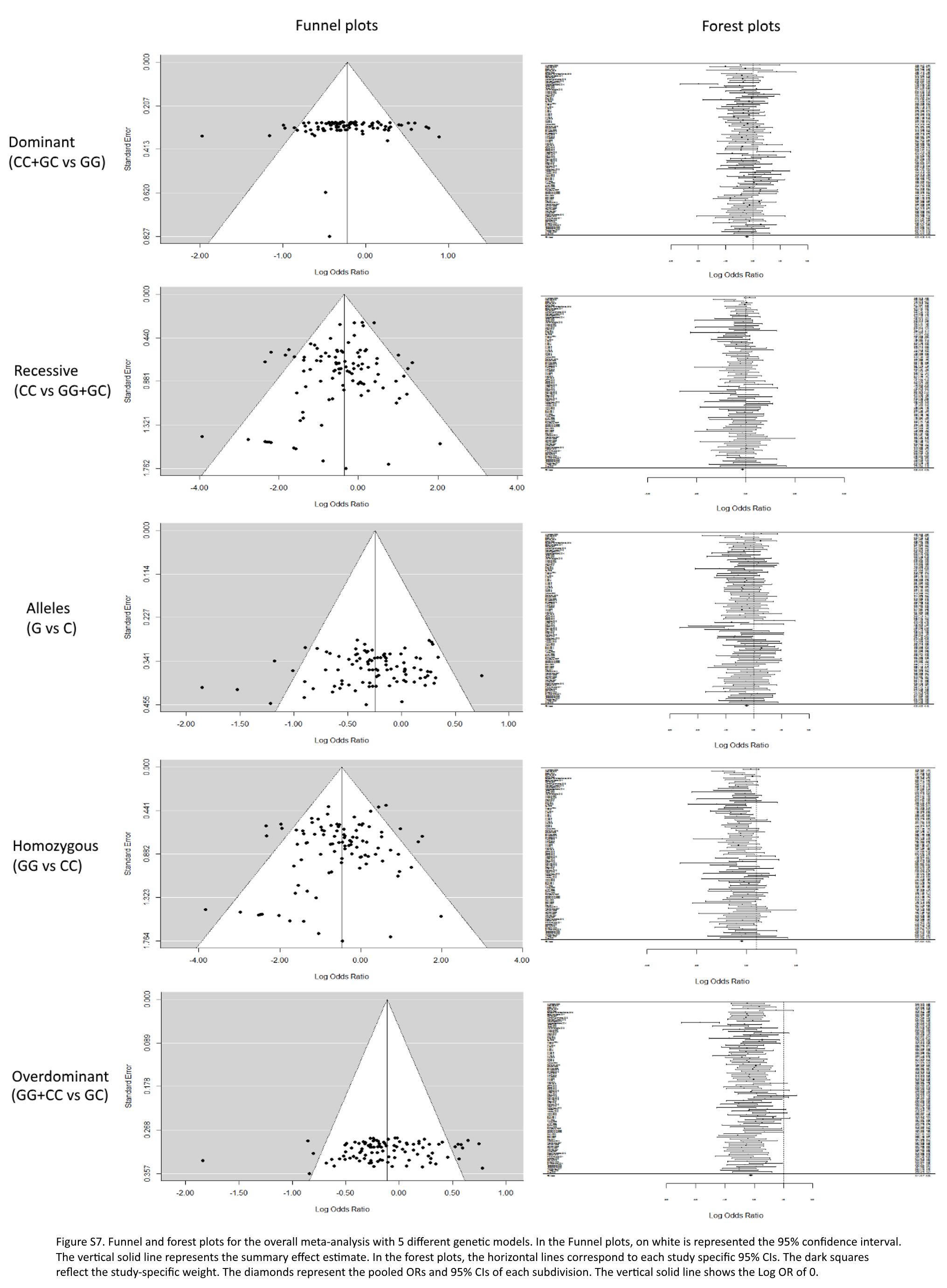

Supplement: Supplementary file 9 [file Image7.TIF]
